# Proximal femoral resection arthroplasty for patients with cerebral palsy and dislocated hips: 20 patients followed for 1–6 years

**DOI:** 10.1080/17453670902804935

**Published:** 2009-02-01

**Authors:** Andreas Knaus, Terje Terjesen

**Affiliations:** Department of Orthopaedic Surgery, Rikshospitalet University Hospital, and Faculty of Medicine, University of OsloOsloNorway

## Abstract

**Background and purpose** Chronic hip dislocation in non-ambulatory individuals with cerebral palsy (CP) can lead to severe problems, of which pain is often the most severe. We studied the outcome of proximal femoral resection, especially regarding pain, sitting balance, perineal care, and patient satisfaction.

**Patients and methods** During the period 1998–2005, we operated 20 non-ambulatory patients with spastic quadriplegic CP (8 females and 12 males). 13 patients had unilateral dislocation and 7 had bilateral. The mean age at operation was 15 (3–27) years. The indications for operation were chronic hip dislocation plus severe problems with pain (17 patients), perineal care (16), and sitting (10). Patients were followed from 1 to 6 years.

**Results** 14 patients were satisfied with the surgery, 3 were dissatisfied, 2 were uncertain, and 1 patient had died 5 days postoperatively. Of the 15 patients who had suffered from considerable pain before surgery, 8 had complete relief from pain and 7 patients experienced improvement. Of the 2 patients who had had mild pain, 1 was unchanged and 1 patient deteriorated. All patients who had not been able to sit were able to sit after the surgery. Only 1 patient had difficulties with perineal hygiene at follow-up. Postoperative complications included deep vein thrombosis (1 patient) and edema, loss of appetite, and the need for gastrostomy (1 patient). 7 patients had prolonged pain for up to 6 months after surgery. 1 of these was reoperated because of persistent pain due to a bony-spike heterotopic ossification.

**Interpretation** Most patients with chronic hip dislocation and severe pain or other major problems appear to benefit from proximal femoral resection. Pain, sitting ability, and perineal care improved and most patients and caregivers were satisfied.

## Introduction

Hip dislocation is not uncommon in patients with cerebral palsy, especially in the most severely retarded and non-ambulatory individuals. Hip dislocation can lead to several problems, of which pain is the most severe. The incidence of pain varies between 25% and 55% (Moreau et al. 1979, Eilert 1997). Sitting function may be affected if hip dislocation is combined with joint contractures and pelvic obliquity. Spastic adductor and flexor muscles and contractures make perineal care difficult.

During the past decade, we have been performing proximal femoral resection interposition arthroplasty (PFRIA) as a salvage procedure in non-ambulatory CP patients with persistent pain, chronic hip dislocation, and markedly reduced function and mobility (Castle and Schneider 1978). We have now evaluated the clinical and radiographic effects of this operation, especially regarding the degree to which the operation reduces pain and improves sitting balance and perineal care.

## Patients and methods

During 1998–2005, we operated 20 non-ambulatory patients (12 male) with spastic quadriplegic CP and chronic hip dislocation with severe problems. The mean age at operation was 15 (3–27) years. The medical charts were reviewed. Criteria for operation were dislocation of the hip that had lasted for more than 2 years and severe problems such as pain, sitting problems, and difficulties with perineal care (Table 1). Most of the patients could not talk; thus, the information on symptoms was usually given by the primary caregiver.

**Table 1.** Clinical data and patient satisfaction

Case	Age	Sex	A	B	C	D	E	F	G	H	I	J
1	20	F	R L	R L	S, PC	T	6	E, LA	51	51	not satisfied	51 m
2	19	F	R L	R L	Pain, PC	T				48	very satisfied	
3	17	F	L	L	S, PC	T	6			55	very satisfied	
4	16	F	L	R L	Pain, S, PC	T				52	uncertain	
5	10	F	L	R L	Pain, S, PC	T						5 d
6	25	M	R	R L	Pain, S	P				27	satisfied	
7	14	F	L	R L	Pain, S, PC	T				40	very satisfied	
8	12	M	L	L	Pain, PC	T				54	satisfied	
9	18	M	L	L	Pain, PC	T	3	DVT		26	very satisfied	
10	13	M	R L	R L	Pain, PC	P				34	satisfied	
11	16	M	R	R L	Pain, PC	T	4–5			72	not satisfied	72 m
12	12	F	R	R L	Pain, PC	P	3			28	very satisfied	
13	11	M	R L	R L	Pain, PC	T				55	very satisfied	
14	27	M	R	R	Pain, PC	N	3			50	satisfied	
15	3	M	R L	R L	S	P				12	very satisfied	19 m
16	16	F	L	L	Pain, PC	P				61	satisfied	
17	14	M	L	R L	Pain, S	T				73	satisfied	
18	15	M	R	R	Pain S	T					not satisfied	3 m
19	12	M	R L	R L	Pain, PC	T				61	very satisfied	
20	12	M	R L	R L	Pain, S, PC	T	6			60	uncertain	

A Side dislocated.

B Side operated on.

C Indication for surgery: S, sitting difficulties; PC, problems with perineal care.

D Postoperative treatment: T, traction; P, plaster; N, no treatment.

E Duration of pain (in months) for at least 3 months and up to 6 months postoperatively.

F Complications: E, edema; LA, loss of appetite; DVT, deep vein thrombosis.

G Time (in months) from primary operation to reoperation.

H Follow-up (months)

I Satisfaction

J Time from operation to death: days (d) or months (m).

13 patients were affected unilaterally and 7 were affected bilaterally. Preoperative anteroposterior radiographs were used to measure the migration percentage (MP), which is the lateral migration of the femoral head (Reimers 1980). If the MP is greater than 33%, the hip is subluxated. An MP of more than 90% means total dislocation. 24 hips were dislocated and 3 were subluxated. 34 femoral resections were performed, bilaterally in 14 patients and unilaterally in 6. The reasons for bilateral operation in 7 of the 13 patients with unilateral dislocation were clinical abnormalities in the hips that were not dislocated (contractures and stiffness), and that we wanted to obtain better symmetry.

The surgical technique was in accordance with McCarthy et al. (1988), including extraperiosteal resection of the proximal femur from 3–4 cm below the lesser trochanter. Interposition technique involved suturing the iliopsoas and gluteal muscles to the hip capsule (“closing” the acetabulum) and covering the femoral stump by suturing the vastus lateralis to the muscles and soft tissues on the medial side. Postoperative treatment included skin traction for 2–5 weeks in 13 patients, skeletal traction with a pin through the proximal tibia in 1 patient, hip spica plaster for 4 weeks in 1 patient, abduction plaster (not including the pelvis) for 2–3 weeks in 4 patients, and no traction or plaster in 1 patient.

Because most patients were severely impaired mentally and functionally, regular follow-up controls could not be performed. A telephone questionnaire was used to provide the necessary clinical information during follow-up. The informants were the patient in 2 cases and the primary caregivers in the remaining 18 (parents in 16 cases and a nurse who knew the patient well in 2). They were asked about the degree of pain: no, mild, or severe pain. Other questions included whether the patient could sit without problems in a wheel-chair and whether they had problems with perineal care. We asked if they were satisfied with the operation, using the alternatives “very satisfied”, “satisfied”, “dissatisfied”, or “uncertain”.

An anteroposterior radiograph was taken on the day after surgery, and compared with those taken preoperatively and at the most recent follow-up examination at the hospital—to assess proximal femoral migration, which was related to the horizontal tangent of the most distal point of the os ischii on the same side (Figure). Pelvic obliquity was measured as the angle between the horizontal line and a line drawn through the most distal points of the left and right os ischii.

Heterotopic bone formation was graded according to McCarthy et al. (1988). Type I is a mushroom-shaped cap of bone over the top of the femoral shaft, type II is a spike of bone on the lateral side of the proximal end of the femur, and type III is a more diffuse ossification between the acetabulum and the femur in the adjacent muscles.

## Results

One patient died 4 days postoperatively, presumably from gastric ulcer with complications, and 1 patient died 3 months postoperatively from unknown causes. The follow-up time for clinical information in the remaining 18 patients was 47 (12–73) months. During the follow-up period 3 patients died, one 19 months postoperatively of unknown causes, one of pneumonia 6 years postoperatively, and one died of respiratory insufficiency 11 days after reoperation for pain from an ectopic bone spike.

**Figure:**
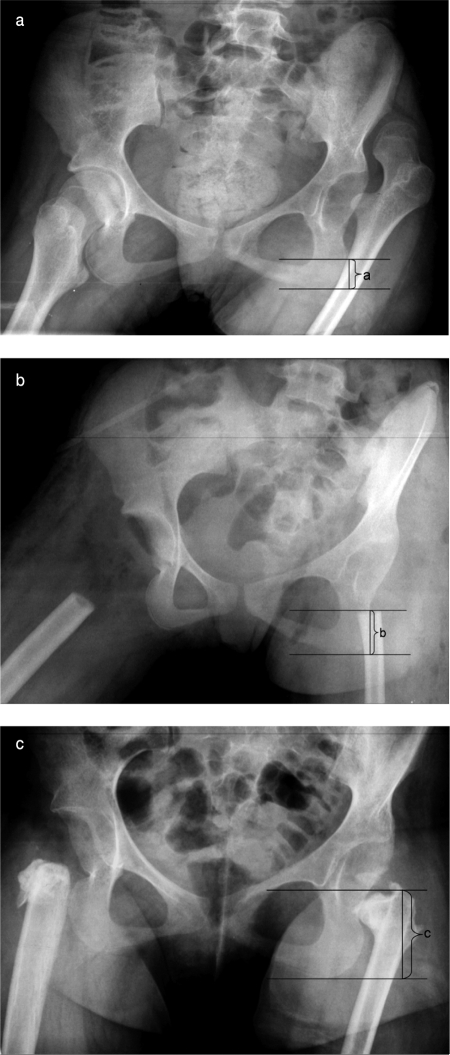
Patient number 7, a 14-year-old girl with quadriplegic cerebral palsy. She had severe pain, was unable to sit in her wheelchair, and had problems with perineal care. A. Radiograph showing left hip dislocation indicating the vertical distance (a) from the femoral resection level to the distal horizontal tangent of the os ischii. B. Radiograph 1 day postoperatively after bilateral PFRIA, showing the vertical distance (b) from the proximal femur to the distal horizontal tangent of the os ischii. C. Radiograph at 16 months, showing the vertical distance (c) from the proximal femur to the distal horizontal tangent of the os ischii and showing bilateral heterotopic ossification.

Preoperatively, 17 patients had experienced severe pain, 2 had had mild pain, and 1 patient had had no pain. Pain status improved in most of the patients with a follow-up of more than 1 year. Of the 15 patients who had suffered from severe pain, 8 experienced complete relief and 7 experienced improvement. Of the 2 patients who had had mild pain, 1 did not experience any change and 1 experienced deterioration (Table 1, no. 1). 1 patient had no pain either preoperatively or postoperatively.

Of the patients with survival of more than 1 year, 8 had had difficulties in sitting before surgery and all of them improved and were able to sit with support at follow-up. Preoperatively, 16 patients had had problems with perineal hygiene. At the time of follow-up, only 1 of these patients had such difficulties.

Complications included prolonged pain lasting more than 3 months and up to 6 months postoperatively in 7 patients, one of which (a girl) was reoperated with resection of the bone spike causing complaints, but she died 11 days after surgery. Of the remaining patients, 4 were free of pain and 2 had mild pain at the latest follow-up. Other complications were deep vein thrombosis (1 patient) and edema, loss of appetite, and need of gastrostomy (1 patient). No patients had wound infections postoperatively.

14 patients were satisfied (8 very satisfied) with the surgery, 3 were dissatisfied, 2 were uncertain, and 1 patient died 5 days postoperatively (Table 1). 1 of the dissatisfied caretakers was the parent of the patient who was reoperated and died. Another dissatisfied patient had pain lasting 4–5 months postoperatively but only mild pain at later follow-up, and he died 6 years after surgery. The third patient died 3 months postoperatively.

The radiographic follow-up was not complete because the 1-day postoperative radiograph was lacking for 2 patients, and in 4 patients later radiographs had not been taken. In the remaining 14 patients, the last pelvic radiograph was taken during a follow-up period of 16 (1–49) months. The proximal femoral migration from preoperatively to the first postoperative day was mean 2.1 cm and the migration was significantly greater in patients with plaster postoperatively (Table 2). The mean migration from the first postoperative day to the last follow-up was 2.9 cm, with no difference between traction and plaster. Total migration on the dislocated side from preoperatively to the last follow-up was 4.8 cm, and the migration was greater in those who had had plaster (6.3 cm) rather than traction (4.0 cm). Total migration was 8.1 (6.1–10) cm in the located hips that had undergone femoral resection.

**Table 2.** Proximal migration (in cm) of the dislocated femur according to postoperative treatment

	Postoperative treatment				p-value	All patients		
	Traction		Plaster **^d^**			Mean	SD	Range
Migration	Mean	SD	Mean	SD				
Postoperative migration **^a^**	1.0	1.3	4.5	1.5	< 0.001	2.1	2.2	-0.9–6.9
From postop. to follow-up **^b^**	3.3	2.1	2.3	1.0	0.4	2.9	1.8	-0.9–6.6
Total migration **^c^**	4.0	1.9	6.3	1.3	0.03	4.8	2.0	1.6–8.0

**^a^** “Postoperative migration” is the migration between the operation and the first postoperative day.

**^b^** “From postop. to follow-up” is the migration from the first postoperative day to the last radiographic follow-up.

**^c^** “Total migration” is the total migration of the femur from preoperatively to the last follow-up.

**^d^** One patient without any postoperative treatment was included in the “Plaster” group.

SD: standard deviation; p-value: the significance level according to Student t-test for independent samples.

Varying amounts of heterotopic ossification developed in all the hips: type 1 in 3, type 2 in 6, and type 3 in 12 hips. The mean pelvic obliquity was 11 (0–33)° before surgery and 6.4 (0–15)° at follow-up (p = 0.1). There was no significant relationship between proximal femoral migration and grade of patient satisfaction (p = 0.2).

## Discussion

In order to avoid hip dislocation, a screening program and early intervention appears to be effective (Hägglund et al. 2005). Prophylactic measures such as adductor tenotomies should be performed (Banks and Green 1960, Terjesen et al. 2005). Soft tissue release should be supplemented by femoral and pelvic osteotomies in the case of severe subluxation or dislocation in children and adolescents (Mubarak et al. 1992, Renshaw et al. 1995). Even so, chronic hip dislocation will develop in some patients.

Chronic dislocation of the hips in non-ambulatory individuals is not in itself an indication for surgery. However, dislocated hips tend to cause increasing problems because of stiffness and joint contractures. The most common complaints—as confirmed in our study—are pain, reduced sitting tolerance, and difficult perineal hygiene (Castle and Schneider 1978, McCarthy et al. 1988, Gamble et al. 1990).

There are very few good treatment options for the non-ambulatory patient with chronic hip dislocation and severe clinical problems. Arthrodesis would not be indicated because this requires being affected unilaterally, weight-bearing function, and no spine involvement (Fucs et al. 2003). Total hip arthroplasty has given satisfactory results in people with CP (Buly et al. 1993, Gabos et al. 1999), but would hardly be indicated in the severely handicapped non-ambulatory patients in our study. We have no personal experience with valgus osteotomy but Hogan et al. (2006) described promising results, although only two-thirds of the caregivers answered the questionnaire about outcome.

The remaining surgical option is femoral resection with soft tissue interposition. Our results confirm the experience from previous studies that this is a fairly reliable procedure in severely spastic non-ambulatory patients with symptomatic hip dislocation (Castle and Schneider 1978, McCarthy et al. 1988, Widmann et al. 1999). According to McCarthy et al. (1988), the procedure is not indicated in growing children because of postoperative pain and proximal migration. In our study, the outcome was not inferior in children under 13 years of age; thus, we do not feel that any specific age limit is necessary.

Pain was considerably reduced at the time of follow-up, which is in accordance with the results of Leet et al. (2005) who reported a reduction in pain from 8.2 to 2.9 using a visual analog scale. Widmann et al. (1999) found that maximal pain relief was achieved at an average of 6 months postoperatively, which is consistent with our experience.

All patients with sitting difficulties were able to sit at the time of the latest follow-up, confirming the results of previous studies that femoral resection provides a marked improvement in sitting ability (Castle and Schneider 1978, McCarthy et al. 1988, Widmann et al. 1999, Abu-Rajab and Bennet 2007). Widmann et al. (1999) found that sitting tolerance improved from 3 h preoperatively to 9 h at follow-up. Almost all of our patients had had problems with perineal hygiene preoperatively, mainly because of adduction contractures. Only 1 patient had such problems during follow-up. This confirms the experience of others (McCarthy et al. 1988, Abu-Rajab and Bennet 2007).

One disadvantage of proximal femoral resection interposition arthroplasty is the relatively long hospital stay because of skeletal or skin traction, in order to avoid proximal migration of the femoral stump. From the first postoperative radiograph to the last follow-up, Leet et al. (2005) found a mean proximal femoral migration of 2.5 cm after postoperative skeletal traction or external fixation, which is similar to what we found. Widmann et al. (1999) found no significant difference in proximal migration between skeletal and skin traction. In our patients, the total migration from preoperatively to last follow-up, which has not been measured in previous studies, was 2.3 cm more after plaster than after skin traction. The clinical significance of migration seems unclear, since Widmann et al. (1999) did not find any correlation between grade of proximal migration and pain and we found no relationship between migration and degree of patient satisfaction.

Postoperative skeletal traction was used by McCarthy et al. (1988), Widmann et al. (1999), and Abu-Rajab and Bennet (2007), while Leet et al. (2005) used skin traction in some patients, skeletal traction in others, and external fixation in yet other patients. The duration of traction varied from 3 and 6 weeks. To ease the postoperative treatment and reduce the hospital stay, we started with abduction plaster. Our preliminary experience in 5 patients indicated that a plaster cast with a broomstick between the legs for 3 weeks to keep the hips moderately abducted is sufficient.

Heterotopic bone formation occurred in all the operated hips. The clinical consequence of ossification is unclear, because most our patients had no complaints from heterotopic bone, which confirms the experience of Abu-Rajab and Bennet (2007).

14 of 19 patients or caregivers were satisfied with the surgery. This result is in accordance with the work of Leet et al. (2005), who reported that 11 of 15 patients or caregivers would recommend the operation to others in a similar situation. Other authors have also reported good results in most patients (McCarthy et al. 1988, Widmann et al. 1999, Abu-Rajab and Bennet 2007).
